# Evaluating the potential of 1-methylcyclopropene treatments on physicochemical properties, bioactive compounds, and shelf life of mango fruits under different storage conditions

**DOI:** 10.1016/j.heliyon.2024.e34695

**Published:** 2024-07-18

**Authors:** Mustafa Kamrul Hasan, Asraful Alam, Md. Rakibul Islam, Md. Akhtaruzzaman, Mrityunjoy Biswas

**Affiliations:** a Department of Agro Product Processing Technology, Jashore University of Science and Technology, Jashore, 7408, Bangladesh; b Department of Food Processing and Preservation, Hajee Mohammad Danesh Science and Technology University, Dinajpur, 5200, Bangladesh

**Keywords:** Mango, 1-MCP, Storage structure, Fruit quality, Storage life

## Abstract

The effect of 1-methylcyclopropene (1-MCP) treatments and storage conditions in the regulation of the physicochemical and bioactive properties of mango fruits (*Mangifera indica* L. cv. BARI-4) were investigated during storage. Different concentrations of 1-MCP treated samples (T_0_ = Control, T_1_ = 0.5 μL/L, T_2_ = 1.0 μL/L, T_3_ = 1.5 μL/L, and T_4_ = 2.0 μL/L) were stored in developed storage structure (10±1 °C and 90 % RH), cold storage (3 ± 1 °C and 80 ± 2 % RH), and ambient storage (29 ± 3 °C and 65 ± 2 % RH). The change in fruit quality including weight loss, firmness, surface color, storage life, chemical, and bioactive properties were studied periodically at 10, 20, 30 and 40 days of storage. The results demonstrated that 1-MCP treatment effectively maintained the quality of fruits by retarding the loss of weight, firmness, total soluble solids (TSS), and titratable acidity, which were served as a quality parameter during storage. The 1-MCP treatment dramatically delayed the change in color, quality measures, and bioactive properties compared to the control group. The storage condition greatly influenced the postharvest quality value and storage life. In combination with the developed storage structure and 1-MCP treatment preserved the acceptability of fruits to a great extent for around 40 days. The T_2_ = 1.0 μL/L 1-MCP treatment preserved the fruit quality for the highest days of storage 14, 34, and 46 days in ambient storage, cold storage, and develop storage structure respectively. The developed storage structure (10 ± 1 °C and 90 % RH) with 1-MCP (1.0 μL/L) treatment suggested the optimum storage ability for preserving the postharvest storage life of mango fruits. By implementing these findings mango growers and suppliers can reduce post-harvest losses, expand market reach, and provide consumers with high-quality mangoes that retain their quality for an extended period.

## Highlights

• Effects of 1-MCP on mangoes' physicochemical and bioactive properties were assessed under three storage conditions.
• Quality parameters and bioactivity were evaluated periodically at 10, 30, and 40 days of storage.
• Storage conditions significantly influence the effectiveness of 1-MCP treatments on the storage life of mango fruits.
• 1-MCP treatments influence physicochemical properties in preserving the quality of mango fruits.
• The developed storage structure and 1-MCP treatment (1.0 μL/L) exhibited the highest storage life of mangoes.

## Graphical abstract

**Figure:**
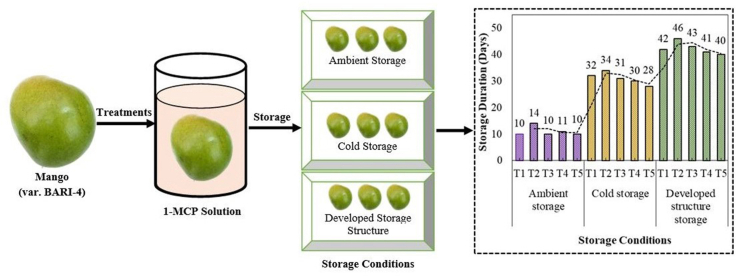

## 1 Introduction

Mango (*Mangifera indica*) is a highly valued tropical fruit for its delicious taste, vibrant color, nutritional value, and health benefits [1]. However, maintaining the fruit’s quality and extending its shelf life poses significant challenges due to its perishable nature. To address this issue, various preservation methods, such as modified atmosphere packaging and postharvest treatments have been explored to inhibit ethylene production, delay ripening, and enhance the storage life of mango [2]. One such postharvest treatment is the application of 1-MCP, which has the ability to inhibit ethylene action. Ethylene is a natural plant hormone responsible for fruit ripening and senescence. By inhibiting ethylene action, 1-MCP effectively delays the ripening process, thereby extending the post-harvest life of mango fruit [3].

The application of 1-MCP involves exposing mango fruits to low concentrations of the compound within a controlled environment, which can occur either before or after harvest [4]. 1-MCP works by binding irreversibly to the ethylene receptor sites, preventing ethylene from binding and initiating the ripening process [5,6]. Consequently, slow down the metabolic activities of fruits leading to extended shelf life, delayed senescence, and better preservation of overall quality attributes. Several studies have investigated the effect of 1-MCP on mango fruits during storage, with promising results [7,8]. One of the key benefits observed in the fruits treated with 1-MCP maintain their texture and firmness for a longer duration compared to untreated fruits [[9], [10], [11]]. The preservation of fruit firmness not only improves consumer acceptance but also reduces the chances of mechanical damage during transportation and handling. Furthermore, the use of 1-MCP has been found to delay the onset of fruit ripening and associated physiological changes. This delay allows for a longer storage period, enabling mangoes to reach distant markets without significant quality deterioration. Studies have reported that 1-MCP-treated mango fruits exhibit reduced rates of fruit softening, color development, and loss of greenness, thereby extending the marketable period [12]. In addition to maintaining firmness and delaying ripening, 1-MCP treatment has been shown to inhibit the development of physiological disorders in mangoes [13]. These disorders include chilling injury, internal breakdown, and senescence-associated browning. By suppressing ethylene’s effects, 1-MCP minimizes the occurrence and severity of these disorders, enhancing the visual appeal and overall quality of the fruits.

The perishable nature of mangoes necessitates careful storage conditions to prolong their shelf life and maintain their quality. Several factors, such as temperature, humidity, and packaging, significantly impact the preservation of fruits during storage [14]. Understanding the effects of these storage conditions is essential for minimizing post-harvest losses and ensuring consumer satisfaction. Temperature plays a crucial role in slowing down physiological processes and retarding the ripening process. Low temperatures, typically between 10 and 13 °C are commonly employed for long-term storage of mangoes. This controlled temperature helps delay fruit ripening and extends their shelf life. The storage temperature below 10 °C fruits are susceptible to chilling injury which appears as peel blackening, discoloration, internal browning, and loss of flavor [15]. Conversely, the higher temperatures above 13 °C accelerate fruit softening, color changes, and deterioration, leading to decreased quality and shelf life. Humidity levels in storage facilities have a profound impact on mango preservation. High humidity environments can lead to excess moisture accumulation, promoting the growth of microorganisms and causing fruit rotting [16]. Conversely, excessively low humidity levels can cause fruit dehydration, resulting in shriveling and loss of flavor [17]. Therefore, maintaining an optimal humidity range is crucial for preserving the firmness, appearance, taste, and other quality parameters of fruits during storage.

Understanding the effects of 1-MCP treatment and different storage conditions on the quality and shelf life of mangoes can provide valuable insights for the mango industry, helping to improve post-harvest practices and maintain the freshness of mangoes during transportation and storage. Several researchers used 1-MCP on different mango varieties to prolong the shelf life. But there is no published article claimed the potentiality of 1-MCP doses along with different storage schemes for improving the shelf life and quality attributes of BARI-4 mango cultivar especially in Bangladesh. Therefore, the aim of this study is to investigate the effect of different concentrations of 1-MCP treatment on mango fruits stored in these three different conditions including developed storage structure, cold storage, and ambient storage. In addition, physicochemical properties, bioactive compounds and overall visual quality was evaluated periodically to assess the impact of 1-MCP treatment and the storage conditions on the mangoes.

## 2 Materials and methods

### 2.1 Sample preparation

Mangoes (*Mangifera indica* L. cv. BARI-4) were harvested manually at commercial maturity when the fruit reaches its mature green stage and starts turning yellow from an orchard in Kotchandpur (Latitude: 23° 39′ 31.07″ N, Longitude: 89° 02′ 08.88″ E), Jhenaidah, Bangladesh. After harvest, mangoes were shifted carefully to the Laboratory of the Department of Agro Product Processing Technology at Jashore University of Science and Technology, Jashore. This process was done in plastic crates, maintaining a temperature of 25–30 °C, humidity of 65–70 %, and within 2–3 h, ensuring proper ventilation and protection from physical damage. The fruit were chosen according to a uniform size (385 ± 10 g) shape, and maturity level at which the fruit exhibit desired physiological and quality attributes for experiment purposes. Afterward, the selected fruit were subjected to hot water treatment at 50^o^C for 5 min for removal of fungal contamination and then air dried to drain off excess water. Thereafter, the fruits were randomly divided into five different categories for further studies.

### 2.2 Brief description of developed refrigerated storage structure

**Fig. 1:**
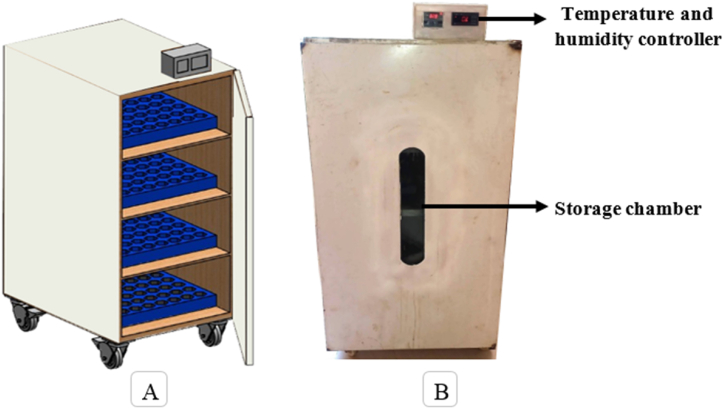
Isometric view (A) and Photographic view of the developed refrigerated storage structure (B).

The refrigerated storage structure was previously developed in the Dept. of Agro Product Processing Technology, Jashore University of Science and Technology, Bangladesh for preserving harvested mango fruit. The dimensions of storage chamber were 0.732 m (2.5 feet) in depth, 0.732 m (2.5 feet) in width and 0.914 m (3.5 feet) in height. The storage structure was constructed with locally available materials such as wood, cork sheet and plastic polymer insulator. The thickness of storage structure wall was 0.0254 m (1 inch). The storage structure was able to regulate within the range of temperature (2–15 °C) and relative humidity (65–95 %) inside the storage chamber. The storage structure was operated possible by single phase (220 V) power source. A digital temperature controller thermostat (Model: XH-W3001, China) and humidity controller (Model: XM-18, China) was set in order to maintain the desired temperature and relative humidity for the preservation. Fig. 1 (A and B) shows the isometric view and photographic view of the developed refrigerated storage structure used in this study.

### 2.3 1-Methylcyclopropane (1-MCP) treatments and storage of fruits

A total of 180 mangoes were allocated for each specified storage conditions as mentioned below. The fruits were then randomly divided into 6 groups, with each group containing 30 fruits, which were used for 5-treatment application. This arrangement resulted in 6 replicates for each treatment. The selected mango fruits were immersed separately in 1-Methylcyclopropene (1-MCP; SmartFresh™), solution for 5 min at different concentrations. The control sample was immersed in water that was preserved without any chemical application. The 5 treatment groups consisted of mangoes that were exposed to 1-MCP application with different doses such as T_0_ = control (without 1-MCP), T_1_ = 0.5 μL/L 1-MCP, T_2_ = 1.0 μL/L 1-MCP, T_3_ = 1.5 μL/L 1-MCP, and T_4_ = 2.0 μL/L 1-MCP according to predefined protocol [18]. After treatment application, the treated and control fruits were stored in three storage environments of different temperatures and relative humidity follows.

i. Developed storage structure (10 ± 1 °C and 90 % RH)
ii. Cold storage (A commercial cold storage having chamber size of (72 × 62 × 6 feet) was used for preservation of mangoes. The temperature and relative humidity were maintained at 3 ± 1 °C and 80 ± 2 % RH, respectively.
iii. Ambient storage (The samples were kept in a separate tray, ensuring proper labeling, in a safe place in the laboratory at 29 ± 3^0^C and 65 ± 2 % RH)

The samples were taken from each target group at 10, 20 30, and 40 days of storage for analysis of physicochemical and bioactive properties.

### 2.4 Physicochemical analysis

#### 2.4.1 Determination of physiological wight loss (PWL), firmness, and total soluble solids (TSS)

The PWL percentage (three fruits for each treated group) was determined for each interval of storage and the results were calculated using the following equation (A-B)/A × 100, where A is the initial weight after treatments before storage and B is the same fruit weight after a specific storage period.

A penetrometer (OSK–I-10576, Ogawa Seiki Co., Tokyo, Japan) equipped with an 8-mm probe was used for the measurement of mango fruit firmness (each time n = 3). Three readings were taken for each mango fruit at different locations after removing peel and the results were expressed in Newton (N).

The TSS levels of mango fruit samples were determined using a digital refractometer (HI 96801 refractometer, China). The results were expressed as ^o^ Brix after the readings were recorded.

#### 2.4.2 Determination of titratable acidity (TA)

The TA of the fruit sample was determined according to the method of [19]. The samples were titrated with 0.1 N NaOH solution using 3–5 drops of phenolphthalein as an indicator. The volume of alkali used was calculated and results were reported as a percentage of citric acid. The TA was determined by using the following equation [1].

(1) $$\text{Acidity}\;\left(\%\right)=\frac{\text{Titre}\;\text{value}\times \text{Normality}\;\text{of}\;\text{alkali}\times 0.06406}{\text{Volume}\;\text{of}\;\text{sample}\;\left(\text{taken}\;\text{for}\;\text{estimation}\right)\;}\times 100$$

#### 2.4.3 Determination of ascorbic acid content (AAC)

The AAC of the fruit sample was estimated by the titration method involving 2,6-dichloroindophenol reduction [20]. In brief, 5 g of sample was homogenized with 50 mL of 2 % oxalic acid, and extract for 20 min. After that, 2 % oxalic acid solution was added to make the final volume 100 mL and filtered through Whatman No. 1 filter paper. Afterward, 10 mL of filtrate was transferred to an Erlenmeyer flask and titrated with 2,6-dichloroindophenol dye to a pink end point persisted for 15 s. The ascorbic acid was determined by using equation [2] as follows.

(2) $$\text{Ascorbic}\;\text{acid}\;\left(\frac{\text{mg}}{100g}\right)=\frac{\text{Titre}\;\text{value}\times \text{Dry}\;\text{factor}\times \text{Volume}\;\text{made}\;\text{up}}{\text{Volume}\;\text{of}\;\text{filtrate}\;\text{taken}\times \text{Weight}\;\text{of}\;\text{sample}}\times 100$$

#### 2.4.4 Determination of total sugar content (TSC)

The TSC of fruit samples was determined using the method described by Lane and Eynon [21]. A quantity of 25 mL lead free filtrate was taken in a 250 mL volumetric flask to which 5 mL concentrated HCL acid was added and mixed properly. Acid was then neutralized with the help of 0.1 N NaOH using phenolphthalein as an indicator till the pink color persisted. After that, the volume was made up to 100 mL and the total sugar content was determined by filling the solution in a burette and titrated against Fehling’s solution. As an indicator methylene blue was used and end point was measured by brick red color. The following equation [3] was used for calculation of percentage total sugars content present in samples.

(3) $$\text{Total}\;\text{sugars}\;\left(\%\right)=\frac{\text{Factor}\times \text{Volume}\;\text{made}\;\text{up}\;}{\text{Titre}\;\text{value}\times \text{Weight}\;\text{of}\;\text{Sample}}\times 100$$

#### 2.4.5 Determination of fruit color

The surface color of the mango sample was evaluated using a precision colorimeter (BCM-110, Biobase, China) to measure the color values such as L* (lightness), a* (greenness), and b* (yellowness). The measurements of color values were taken in three locations of each mango fruit at 10, 20, 30, and 40 days of storage.

### 2.5 Antioxidants analysis

#### 2.5.1 Extraction of bioactive compounds

The extracts were prepared from mango fruit pulp according to the procedure [22]. In details, 10 g of samples were homogenized in 20 mL of 95 % ethanol using a homogenizer for 5 min. After that, the mixture was centrifuged at 3000 rpm for 5 min. Repetition of the extraction process was done after collecting the supernatant. The samples were then again dissolved in 95 % ethanol while being stirred, yielding a final volume of 20 mL and a final concentration of 1 g fresh weight/kg. Finally, the extracts were kept at −20 ^o^C for further research.

#### 2.5.2 Determination of total phenolic content (TPC)

The TPC value of mango fruit extract was measured using a modified Folin-Ciocalteu technique [23]. In brief, 1 mL of fruit extracts were mixed with 4 mL of freshly prepared Folin-Ciocalteu reagent (1:10 v/v in distilled water). After that, 1 mL of sodium carbonate (7.5 % solution, w/v) was added and vortexed for 15 s in order to develop the color. Afterward, the mixture was then allowed to stand at 40 °C for 30 min. Thermo Scientific’s double-beam UV–Vis spectrophotometer (T60, UV–Vis Spectrophotometer, USA) was used to measure the absorbance at 765 nm. Gallic acid was used as standard and distilled water was used to prepare the blank in replace of the sample. The values of TPC was expressed as mg of gallic acid equivalent per kg of fresh weight (mg GAE/kg FW).

#### 2.5.3 Determination of total flavonoid content (TFC)

The amount of TFC in extracts was determined using colorimetric method [24]. In brief, 5 mL of ethanolic extract was taken and combined with 2.5 mL of AlCl_3_ reagent, which was prepared by the addition of 133 mg of crystalline AlCl_3_ and 400 mg of crystalline sodium acetate in 100 mL of deionized water. The absorbance was measured using the UV–Vis spectrophotometer (T60, UV–Vis Spectrophotometer, USA) at 430 nm followed by 15 min incubation at room temperature. The results were expressed as mg of quercetin equivalent per kg of fresh weight (mg QE/kg FW) using quercetin as standard.

### 2.6 Scanning electron microscopic (SEM), and storage life analysis

The SEM studies of the mango samples were carried out using Leo surface scanning electron microscope (Model 435VP, Leo Electronic Systems, Cambridge, UK). The surface microstructure of control and treated (T_2_ and T_3_) mango samples were examined to compare the effectiveness of 1-MCP at different concentrations with a 500x magnification.

The marketability of fruits was subjectively evaluated by observing the level of visible mold growth, decay, shriveling, smoothness, and shine of fruits, these descriptive quality attributes were determined subjectively.

### 2.7 Statistical analysis

The obtained data was analyzed statistically using SPSS software (version 25.0). The analysis of variance (ANOVA) was conducted to determine the significance level of all parameters, and Tukey’s range test was utilized to determine the significant difference between means at p ≤ 0.05. The regression analysis was performed by using RStudio version 4.0.3 [25].

## 3 Results and discussion

### 3.1 Physiological weight loss (PWL)

PWL is considered the major index of post-harvest storage life and quality of fresh fruits. It is mainly attributed to water loss through the process of respiration and transpiration, which reduces the quality of fruits by causing them to shrink, wrinkle, and weight loss [26]. The PWL of BARI-4 mangoes treated with different concentrations of 1-MCP and untreated control samples stored in three different conditions have been shown in Table 1. The treated sample exhibited lower water loss than untreated control samples. This was because 1-MCP treated fruit surface retard oxygen, carbon dioxide, and moisture migration thus reducing ethylene production, oxidation reaction, and PWL [27].

**Table 1:** Interaction effects of 1-MCP treatments on changes in the weight loss and firmness in developed storage, cold storage and ambient storage conditions.

Storage condition	Treatment	Weight loss (%)				Firmness (N)			
		Storage period (days)				Storage period (days)			
		10	20	30	40	10	20	30	40
Developed storage structure (10±1 °C and 90 % RH)	T_0_	7.62 ± 0.69^gh^	13.8 ± 0.66^a^	18.83 ± 0.27^a^	23.85 ± 0.82^a^	14.26 ± 0.74^g^	7.12 ± 0.42^de^	2.29 ± 0.69^d^	0.31 ± 0.15^c^
	T_1_	2.88 ± 0.30^gh^	8.55 ± 0.33^b^	16.08 ± 0.35^b^	19.88 ± 0.57^b^	18.96 ± 0.65^d^	9.69 ± 0.67^c^	5.05 ± 0.35^c^	0.96 ± 0.28^c^
	T_2_	2.05 ± 0.43^h^	4.61 ± 0.34^e^	9.39 ± 0.59^e^	13.45 ± 0.80^d^	26.93 ± 0.44^a^	16.58 ± 0.64^b^	5.51 ± 0.70^bc^	4.93 ± 0.49^a^
	T_3_	2.16 ± 0.42^gh^	5.17 ± 0.24^de^	10.74 ± 0.55^de^	15.05 ± 0.68^cd^	26.48 ± 0.51^a^	19.48 ± 0.62^a^	8.88 ± 0.71^a^	5.02 ± 0.41^a^
	T_4_	3.47 ± 0.33^g^	6.33 ± 0.53^cd^	11.83 ± 0.72^d^	16.65 ± 0.57^c^	24.27 ± 0.83^b^	15.76 ± 0.53^b^	7.03 ± 0.76^b^	2.66 ± 0.45^b^
Cold storage (3±1 °C and 80 ± 2 % RH)	T_0_	5.72 ± 0.29^f^	8.04 ± 0.43^b^	18.60 ± 0.60^a^	NA	17.00 ± 0.71^ef^	2.73 ± 0.51^f^	0.39 ± 0.08^e^	NA
	T_1_	3.16 ± 0.58^f^	7.02 ± 0.50^b^	16.80 ± 0.79^b^	NA	15.81 ± 0.73^g^	7.34 ± 0.70^de^	1.20 ± 0.41^de^	NA
	T_2_	2.36 ± 0.34^gh^	5.21 ± 0.45de	14.29 ± 0.39c	NA	22.35 ± 0.61^c^	6.24 ± 0.68^e^	2.54 ± 0.40^d^	NA
	T_3_	2.48 ± 0.30^gh^	6.55 ± 0.59^c^	14.46 ± 0.34^c^	NA	19.75 ± 0.47^d^	7.69 ± 0.68^de^	4.71 ± 0.49^c^	NA
	T_4_	2.98 ± 0.32^g^	6.57 ± 0.30c	15.61 ± 0.52^bc^	NA	18.13 ± 0.38^de^	8.21 ± 0.50^cd^	1.96 ± 0.27^d^	NA
Ambient storage (29±3 °C and 65 ± 2 % RH)	T_0_	20.13 ± 0.53^a^	NA	NA	NA	0.29 ± 0.24^i^	NA	NA	NA
	T_1_	19.26 ± 0.33^a^	NA	NA	NA	1.45 ± 0.59^i^	NA	NA	NA
	T_2_	9.27 ± 0.66^d^	NA	NA	NA	1.20 ± 0.45^i^	NA	NA	NA
	T_3_	11.00 ± 0.43^c^	NA	NA	NA	3.84 ± 0.69^h^	NA	NA	NA
	T_4_	16.78 ± 0.67^b^	NA	NA	NA	1.79 ± 0.59^i^	NA	NA	NA

Each value is expressed as mean ± SD and different small letters within the same column indicate significant (p ≤ 0.05) differences among various samples. Note: NA = Not applicable; T_0_ = control, T_1_ = 0.5 μL/L 1-MCP, T_2_ = 1.0 μL/L 1-MCP, T_3_ = 1.5 μL/L 1-MCP, and T_4_ = 2.0 μL/L 1-MCP treatment.

The PWL of mango samples increased with the advancement of storage time, rather slowly in the beginning but faster with the advance of the storage period. The data presented in Table 1 displayed that, PWL of mango sample in all treatments were increased significantly with the increase in storage duration. This findings are in agreement with Li et al. [12] and Sakhale et al. [28], who observed similar behavior of mango fruits treated with different concentration of 1-MCP. At initial days of storage (10 days), the lowest PWL was observed in (T_2_ = 2.05 ± 0.43 %) fruits treated with 1-MCP at a concentration of 1.0 μL/L and stored in developed storage structure at 10±1 °C and 90 ± 1 % RH, which was found to be statistically significant as compared to other treatments. On the other hand, the highest PWL was observed (T_0_ = 20.13 ± 0.53 %) in control sample stored in ambient condition 29 ± 3^0^C and 85 ± 2 % RH. The storage conditions had significantly (p < 0.05) influenced the PWL of fruits for both treated and control samples. Therefore, at final day of storage (40 days), the highest and lowest PWL found in developed storage condition from T_0_ (23.85 ± 0.57 %) and T_2_ (13.85 ± 0.80 %) respectively, indicating that the storage conditions in developed storage structure (10 ± 1 °C and 90 % RH) with 1-MCP treatment was much more effective in reducing PWL and retaining freshness of mango fruits. Whereas, at 10 days of ambient storage and 30 days of cold storage fruits lose their natural freshness and quality due to rapid moisture loss.

### 3.2 Firmness

The firmness of fruits is mostly determining its postharvest storage life, freshness and quality [29]. Interaction between different concentration of 1-MCP treatments and storage conditions had a significant impact (p < 0.05) on the firmness change of BARI-4 mango samples have been shown in Table 1. The treated samples maintained higher firmness as compared to control samples at storage intervals. During the 10-days of storage, the significantly highest firmness retention in fruits was observed in T_2_ (26.93 ± 0.44 N) and T_3_ (26.48 ± 0.51 N) stored at developed storage structure, which were treated with 1-MCP at a concentration of 1.0 μL/L and 1.5 μL/L respectively. Whereas, the lowest firmness value T_0_ = 0.29 ± 0.24 N was found in control sample stored in ambient condition. The storage conditions used in developed storage structure exhibited higher firmness retention as compared to cold storage and ambient storage conditions. The effect of 1-MCP treatment on fruit firmness was largely influenced by the storage condition [9].

Therefore, at final day of storage (40 days), the firmness change was recorded in developed storage condition at 10 ± 1 °C and 90 ± 1 % RH. The highest firmness was found in fruits treated with 1-MCP at a concentration of T_3_ = 1.5 μL/L, while least firmness was observed in control treatment (T_0_) and the values were 5.02 ± 0.41 N and 0.31 ± 0.15 N respectively. Similarly, as PWL of fruits sample, after 10 days of ambient storage and 30 days of cold storage fruit samples did not retain its natural freshness and quality. This findings are in agreement with Li et al. [12], who found negligible firmness change of mango fruits treated with 1-MCP at a concentration of 1.0 μL/L from 6 to 10 days of storage at 25 °C. However, the trends in firmness retention capacity during storage were the order of developed storage structure > cold storage > ambient storage.

The firmness of the treated and untreated control samples in different storage conditions decreased due to the softening of fruit tissue with the enhancement of storage duration. The softening of fruit tissue is normally attributed to the destruction of cell structure and deterioration of cell wall composition caused by the biochemical process that involves the hydrolysis of starch and pectin by enzymes such as pectinesterase and polyglacturonase [26,27]. It was evident that, treatment with 1-MCP could inhibit the activity of enzyme related to softening of fruits in the ripening process thus inhibiting the degradation of cell structure [30].

### 3.3 Total soluble solids (TSS)

The number of dissolved solids present in fruits juice is determined by TSS, that indicate the sweetness and ripeness of fruits. It is crucial to monitor the TSS of fruits so that the fruits is harvested at the appropriate stage of ripeness to ensure its sustainability for storage or further processing [29]. The change in TSS value of treated and control samples has been displayed in Table 2. The storage time and the interaction effects of 1-MCP treatments with storage conditions had significantly (p < 0.05) affect the TSS values of the fruit samples. This was clearly observed from the results, at initial storage (10 days) the highest TSS was detected in control sample (T_0_) stored in ambient storage and the lowest TSS was found in the 1-MCP treated sample (T_2_) at a concentration of 1.0 μL/L in cold storage and the values were 21.45 ± 0.29 and 10.35 ± 0.30 °Brix respectively. However, the highest value found from the T_2_ stored in cold storage (3 ± 1 °C and 80 ± 2 % RH), which was not significantly different from the treatment T_2_ in developed storage structure (10 ± 1 °C and 90 ± 1 % RH) and the value was 10.46 ± 0.31 °Brix. The results found in this research were close approximately with results obtained by Ref. [28]. During the first 10 days of storage, the increase in TSS value of treated and control samples at ambient storage could be due to excessive moisture loss as well as the hydrolysis of carbohydrates to soluble sugars [31]. This may attribute to the higher storage temperature and lower relative humidity influenced in faster conversion of starch into water-soluble sugars resulting in a shorter shelf life.

**Table 2:** Interaction effects of 1-MCP treatments on changes in the total soluble solids (°Brix) and total sugar content in developed storage, cold storage, and ambient storage conditions.

Storage condition	Treatment	Total soluble solids (^o^Brix)				Total sugar content (%)			
		Storage period (days)				Storage period (days)			
		10	20	30	40	10	20	30	40
Developed storage structure (10±1 °C and 90 % RH)	T_0_	16.80 ± 0.65^c^	14.50 ± 0.69^bc^	16.96 ± 0.32^bc^	18.53 ± 0.53^a^	10.29 ± 2.55^efg^	14.72 ± 0.54^b^	17.58 ± 0.73^c^	17.02 ± 0.27^a^
	T_1_	12.67 ± 0.44^ef^	10.53 ± 0.44^g^	14.26 ± 0.52^ef^	15.73 ± 0.51^bc^	8.97 ± 9.41^fgh^	10.50 ± 0.55^d^	14.34 ± 0.49^d^	15.93 ± 0.73^ab^
	T_2_	10.46 ± 0.31^g^	11.60 ± 0.42^fg^	13.32 ± 0.53^f^	14.61 ± 0.54^c^	8.80 ± 8.76^gh^	9.323 ± 0.48^d^	11.73 ± 0.79^f^	13.16 ± 0.50^d^
	T_3_	11.55 ± 0.63^fg^	14.41 ± 0.28^bcd^	14.38 ± 0.43^ef^	12.88 ± 0.59^d^	10.33 ± 3.37^efg^	10.70 ± 0.94^d^	12.75 ± 0.26^ef^	14.30 ± 0.54^cd^
	T_4_	13.62 ± 0.56^de^	10.38 ± 0.19^g^	15.12 ± 0.50^de^	16.01 ± 0.38^b^	9.38 ± 3.73^fgh^	12.65 ± 0.17^c^	13.36 ± 0.15^de^	15.16 ± 0.61^bc^
Cold storage (3±1 °C and 80 ± 2 % RH)	T_0_	12.62 ± 0.55^ef^	18.68 ± 0.90^a^	20.38 ± 0.42^a^	NA	10.48 ± 4.58^ef^	16.65 ± 0.48^a^	23.71 ± 0.59^a^	NA
	T_1_	10.72 ± 0.45^g^	15.25 ± 0.37^b^	18.12 ± 0.25^b^	NA	8.26 ± 2.37^h^	16.44 ± 0.70^a^	21.35 ± 0.16^b^	NA
	T_2_	10.35 ± 0.30^g^	12.59 ± 0.54^ef^	15.09 ± 0.57^de^	NA	11.69 ± 6.55^e^	12.44 ± 0.09^c^	17.33 ± 0.62^c^	NA
	T_3_	11.26 ± 0.58^fg^	13.02 ± 0.43^def^	16.35 ± 0.40^cd^	NA	8.31 ± 3.57^h^	13.73 ± 0.78^bc^	18.57 ± 0.28^c^	NA
	T_4_	10.49 ± 0.79^g^	13.25 ± 0.14^cde^	17.80 ± 0.26^b^	NA	14.00 ± 0.63^d^	15.30 ± 0.25^ab^	18.31 ± 0.52^c^	NA
Ambient storage (29±3 °C and 65 ± 2 % RH)	T_0_	21.43 ± 0.29^a^	NA	NA	NA	23.47 ± 4.42^a^	NA	NA	NA
	T_1_	19.64 ± 0.42^b^	NA	NA	NA	21.01 ± 0.45^b^	NA	NA	NA
	T_2_	14.83 ± 0.53^d^	NA	NA	NA	17.43 ± 4.38^c^	NA	NA	NA
	T_3_	17.86 ± 0.62^c^	NA	NA	NA	18.79 ± 7.69^c^	NA	NA	NA
	T_4_	17.63 ± 0.50^c^	NA	NA	NA	18.33 ± 3.28^c^	NA	NA	NA

Each value is expressed as mean ± SD and different small letters within the same column indicate significant (p ≤ 0.05) differences among various samples. Note: NA = Not applicable; T_0_ = control, T_1_ = 0.5 μL/L 1-MCP, T_2_ = 1.0 μL/L 1-MCP, T_3_ = 1.5 μL/L 1-MCP, and T_4_ = 2.0 μL/L 1-MCP treatment.

Therefore, the effect of developed storage structure was found to be significant (p < 0.05) during 40 days of storage period. It was evident from the data that developed storage structure maintained TSS with slight increasing trends for both treated and control samples. The retention of TSS values might be due to the slower respiration rate and weaker metabolic activity, thus resulting in delay ripening process [32].

### 3.4 Total sugar content (TSC)

The accumulation of TSC in fruits significantly increased with the advancement of ripening period irrespective to treatment applied [33]. The application of 1-MCP treatment at different concentration and storage conditions delayed the accumulation of TSC compared to control treatment but in some cases the values were not differ significantly from control treatment and the results have been shown in Table 2. The amount of TSC in fruit samples among the three storage conditions exhibited an increasing tendency, as the increase in TSS represents the ability of different concentration of 1-MCP to retard biochemical process associated with fruit ripening. It was obvious from the result that, the higher sugar content was detected for the control treatment T_0_ = 23.47 ± 4.42 % in the ambient storage condition and lower for the treatment of T_1_ = 8.26 ± 2.37 % in cold storage during 10 days of storage. The 1-MCP treated samples exhibited lower levels of TSC compared to control treatment suggests that the formation of a semipermeable barrier by 1-MCP is a consequence of the binding of its molecules to receptors on the fruit surface thus inhibiting ethylene perception and delayed ripening process. These findings are in agreed with Maqbool et al. [27], who found similar results from banana fruits treated with gum arabic in combination with chitosan at 13 ± 1 °C and 80 ± 3 % RH. A similar behavior was found in strawberry fruit treated with 1-MCP accumulated less TSC than control samples, suggesting a role of ethylene in sugar accumulation [34]. The results obtained at the ripened stage (40 days) expressed that with the increase of storage duration the TSC increased. The possible explanation for increase in TSC during storage of mango fruits may possible due to the breakdown of complex organic metabolites into simple molecules [35], or hydrolysis of starch into sugar [36]. Therefore, the treatment T_2_ exhibited most effective treatment and showed lower sugar value compared to other treatments at developed storage condition.

### 3.5 Titratable acidity (TA)

Assessing the acidity level of fruits is a crucial aspect in evaluating their quality and acceptability [9]. The TA content decreased with the advance of storage period but no significant differences were observed between control and 1-MCP treated samples (Table 3). This coincident with results were in agreement with Massolo et al. [10], who did not found significant difference during storage of 1-MCP treated eggplant fruits. However, the TA content of 1-MCP treated samples remained at a slightly higher amount than control sample stored in three different conditions at initial storage. While extending the storage duration, the gradual decrease in terms of TA was found for both cold storage and developed storage structure. The reduction of TA level can be associated with the presence of higher respiration rate results in degradation of organic acids. Thus, the organic acid can act as a substrate for the enzymatic process of respiration leading to a decrease in acidity [27]. Therefore, it was evident that 1-MCP treatment can diminish ethylene action leading to the prevention of O_2_ and moisture migration from fruits and reduce respiration that can prevent acid oxidation.

**Table 3:** Interaction effects of 1-MCP treatments on changes in the titratable acidity and ascorbic acid content in developed storage, cold storage, and ambient storage conditions.

Storage condition	Treatment	Titratable acidity (%)				Ascorbic acid content (mg/100 g)			
		Storage period (days)				Storage period (days)			
		10	20	30	40	10	20	30	40
Developed storage structure (10±1 °C and 90 % RH)	T_0_	0.69 ± 0.07^ab^	0.55 ± 0.22^a^	0.48 ± 0.08^a^	0.47 ± 0.08^a^	29.7 ± 0.62^b^	21.06 ± 0.30^c^	20.9 ± 0.98^c^	14.00 ± 0.31^a^
	T_1_	0.65 ± 0.07^ab^	0.43 ± 0.07^a^	0.47 ± 0.04^a^	0.42 ± 0.08^a^	29.0 ± 0.46^bc^	23.25 ± 0.58^ab^	22.9 ± 0.94^ab^	10.24 ± 0.48^b^
	T_2_	0.38 ± 0.04^b^	0.42 ± 0.18^a^	0.38 ± 0.05^a^	0.31 ± 0.09^a^	28.3 ± 0.41^c^	23.42 ± 0.52^ab^	22.9 ± 0.92^ab^	9.83 ± 0.36^b^
	T_3_	0.49 ± 0.05^ab^	0.48 ± 0.13^a^	0.45 ± 0.17^a^	0.35 ± 0.10^a^	29.1 ± 0.34^bc^	23.80 ± 0.52^a^	24.2 ± 0.26^a^	10.50 ± 0.44^b^
	T_4_	0.56 ± 0.15^ab^	0.48 ± 0.13^a^	0.41 ± 0.11^a^	0.36 ± 0.14^a^	28.8 ± 0.29^bc^	22.19 ± 0.52^bc^	22.2 ± 0.27^bc^	14.39 ± 0.38^a^
Cold storage (3±1 °C and 80 ± 2 % RH)	T_0_	0.72 ± 0.13^ab^	0.52 ± 0.06^a^	0.46 ± 0.12^a^	NA	31.1 ± 0.32^a^	9.360 ± 0.28^g^	9.15 ± 0.15^d^	NA
	T_1_	0.57 ± 0.05^ab^	0.48 ± 0.09^a^	0.45 ± 0.14^a^	NA	27.8 ± 0.46^c^	11.12 ± 0.41^ef^	7.48 ± 0.48^e^	NA
	T_2_	0.54 ± 0.07^ab^	0.55 ± 0.29^a^	0.41 ± 0.21^a^	NA	25.1 ± 0.35^d^	12.08 ± 0.34^de^	5.08 ± 0.08^f^	NA
	T_3_	0.47 ± 0.17^ab^	0.49 ± 0.07^a^	0.49 ± 0.07^a^	NA	27.9 ± 0.51^c^	13.05 ± 0.45^d^	6.89 ± 0.89^e^	NA
	T_4_	0.55 ± 0.10^ab^	0.53 ± 0.17^a^	0.50 ± 0.05^a^	NA	28.0 ± 0.48^c^	10.62 ± 0.56^fg^	7.49 ± 0.49^e^	NA
Ambient storage (29±3 °C and 65 ± 2 % RH)	T_0_	0.81 ± 0.13^a^	NA	NA	NA	9.11 ± 0.49^e^	NA	NA	NA
	T_1_	0.44 ± 0.17^ab^	NA	NA	NA	7.65 ± 0.42^f^	NA	NA	NA
	T_2_	0.59 ± 0.20^ab^	NA	NA	NA	5.10 ± 0.25^g^	NA	NA	NA
	T_3_	0.46 ± 0.12^ab^	NA	NA	NA	6.99 ± 0.33^f^	NA	NA	NA
	T_4_	0.55 ± 0.17^ab^	NA	NA	NA	7.58 ± 0.56^f^	NA	NA	NA

Each value is expressed as mean ± SD and different small letters within the same column indicate significant (p ≤ 0.05) differences among various samples. Note: NA = Not applicable; T_0_ = control, T_1_ = 0.5 μL/L 1-MCP, T_2_ = 1.0 μL/L 1-MCP, T_3_ = 1.5 μL/L 1-MCP, and T_4_ = 2.0 μL/L 1-MCP treatment.

### 3.6 Ascorbic acid content (AAC)

AAC of fruits is one of the most important nutritional quality factors, which may continue its losses during postharvest storage associated with the deterioration of quality. However, these losses can be minimized within certain limits by the application of various postharvest treatments [37]. The AAC of both untreated control and 1-MCP treated samples notably decreased during storage as shown in Table 3. The maximum AAC (31.1 ± 0.32 mg/100 g) was found in (T_0_) control fruits on 10 days of cold storage condition, while in 1-MCP treated fruits the raise of AAC was delayed due to suppressed ethylene action associated with ripening rate. These findings are in agreed with Sachin et al. [35], who found similar behavior of 1-MCP treated guava fruits during low temperature storage. Afterward, the retention of AAC was significantly (p < 0.05) higher in treated sample than untreated control sample after 20 days of storage and continues up to 30 days of storage. The decrease in AAC in 1-MCP treated mango fruit at 10 days may be attributed to the initial impact of the treatment on metabolic processes, causing a temporary decline. However, the subsequent increase in AAC at 20 days could be a result of the fruit’s adaptive response, potentially triggering a compensatory mechanism or enhanced synthesis of ascorbic acid over time. It was evident that the 1-MCP treated mango fruits retain higher vitamin C content which might be due to the capability of accumulation of active oxygen species leading to prevent vitamin C loss [38]. At initial storage, the retention of AAC was higher in cold storage condition compared to ambient storage and developed storage structure. This could be for the reason that low temperature retards postharvest changes through the reduction of respiration rate and other undesirable metabolic changes. Therefore, after 20 days of storage the developed storage structure exhibited better performance on retention of AAC. Whereas, 1-MCP treatment did not affect the retention of ascorbic acid content significantly (p < 0.05) during 40 days of storage compared to control sample.

### 3.7 Surface color

**Fig. 2:**
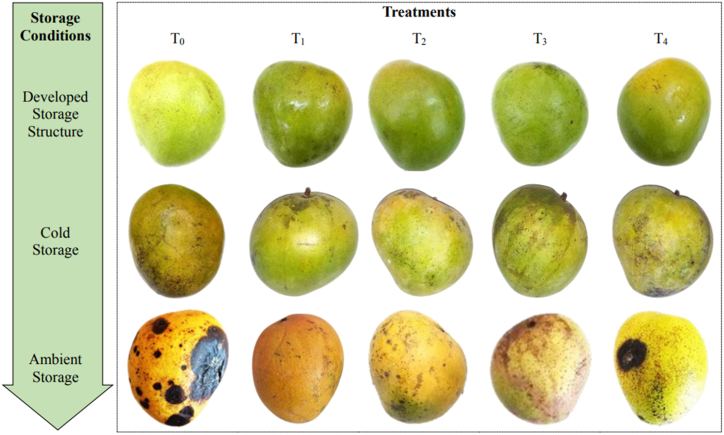
The visual appearance of mango fruits after 1-MCP treatment at 10 days of storage. Note: T_0_ = control, T_1_ = 0.5 μL/L 1-MCP, T_2_ = 1.0 μL/L 1-MCP, T_3_ = 1.5 μL/L 1-MCP, and T_4_ = 2.0 μL/L 1-MCP treatment.

The change in surface color is an important criterion in determining postharvest quality and consumer acceptability, especially of mango fruits [27]. Accordingly, the change in color parameters such as L*, a*, and b* values of control and 1-MCP treated mango fruits stored in developed storage structure, cold storage, and ambient storage were measured and were shown in Table 4. The noticeable increase in L* and b* values were observed in mango samples suggested that the color of fruit epidermis become lighter and turns yellow. This findings were consisted with Yuan et al. [8], who noticed similar activities in mango fruits treated with 1-MCP and in combination with 1-MCP and melatonin. The storage conditions had significant (p < 0.05) effect on preserving the color of fruits. The highest rate of color change especially L* and b* values were observed in ambient storage and cold storage suggesting that developed storage structure effectively preserving the freshness and quality of mango samples. Fig. 2 represented the overall visual appearance of treated and control fruits during 10 days of storage. In line with the results of this study, the use of 1-MCP treatment and storage condition showed positive effect on color retention of mango fruits as shown in Fig. 2. However, the ability of preserving the color parameters of fruit samples were in the trends of developed storage structure > cold storage > ambient storage.

**Table 4:** Effect of 1-MCP treatment on surface color L*, a*, and b* value of mango fruits during storage at developed storage, cold storage, and ambient storage conditions.

Storage Condition	Treatment	Surface color of fruits											
		L*				a*				b*			
		10	20	30	40	10	20	30	40	10	20	30	40
Developed storage structure (10±1 °C and 90 % RH)	T_0_	26.41 ± 0.21^g^	29.05 ± 0.4^f^	48.05 ± 0.36^d^	59.99 ± 0.6^a^	1.49 ± 0.69^defg^	4.46 ± 0.54^a^	2.44 ± 0.21^c^	3.31 ± 0.21^b^	15 ± 0.47^e^	14.9 ± 0.57^c^	32.57 ± 0.15^ef^	36.4 ± 0.19^b^
	T_1_	15.29 ± 0.76^j^	26.39 ± 0.38^g^	48.28 ± 0.66^d^	59.6 ± 0.26^a^	0.33 ± 0.62^fg^	2.16 ± 0.66^def^	4.56 ± 0.4^a^	4.97 ± 0.55^a^	1.98 ± 0.37^h^	15.5 ± 0.4^c^	35.44 ± 0.55^cd^	39.42 ± 0.06^a^
	T_2_	15.8 ± 0.56^j^	36.47 ± 0.38^c^	35.77 ± 0.62^f^	43.14 ± 0.48^d^	−1.63 ± 0.50^h^	1.32 ± 0.16^f^	1.84 ± 0.24^c^	3.46 ± 0.3^b^	2.11 ± 0.43^h^	11.97 ± 0.47^e^	31.33 ± 0.57^f^	33.74 ± 0.25^d^
	T_3_	16.43 ± 0.58^j^	29.64 ± 0.62^f^	39.33 ± 0.41^e^	55.7 ± 0.41^b^	3.51 ± 0.59^abc^	4.14 ± 0.91^ab^	2.1 ± 0.25^c^	5.75 ± 0.32^a^	5.24 ± 0.69^g^	13.21 ± 0.36^de^	32.31 ± 0.38^ef^	36.15 ± 0.17^b^
	T_4_	19.51 ± 0.18^i^	26.4 ± 0.65^g^	35.75 ± 0.48^f^	52.37 ± 0.67^c^	2.14 ± 0.57^cde^	2.93 ± 0.21^bcde^	2.32 ± 0.54^c^	4.43 ± 0.85^ab^	3.88 ± 1.21^g^	13.35 ± 0.55^d^	33.06 ± 0.44^e^	35.49 ± 0.03^c^
Cold storage (3±1 °C and 80 ± 2 % RH)	T_0_	28.78 ± 0.6^f^	44.7 ± 0.46^a^	59.51 ± 0.55^a^	NA	3.32 ± 0.86^abc^	1.68 ± 0.27^ef^	3.74 ± 0.28^ab^	NA	39.78 ± 0.46^a^	32.38 ± 0.36^b^	39.42 ± 0.18^a^	NA
	T_1_	22.84 ± 0.49^h^	39.51 ± 0.23^b^	59.26 ± 0.9^a^	NA	1.34 ± 0.56^efg^	3.23 ± 0.46^abcd^	3.98 ± 0.5^a^	NA	4.4 ± 0.49^g^	35.57 ± 0.43^a^	36.45 ± 0.52^bc^	NA
	T_2_	15.86 ± 0.34^j^	31.7 ± 0.43^e^	52.51 ± 0.77^c^	NA	0.12 ± 0.71^gh^	1.82 ± 0.46^ef^	2.72 ± 0.21^bc^	NA	11.41 ± 0.69^f^	31.49 ± 0.49^b^	34.47 ± 0.51^d^	NA
	T_3_	22.99 ± 0.55^h^	34.81 ± 0.43^d^	60.94 ± 0.6^a^	NA	2.26 ± 0.62^bcde^	2.68 ± 0.5^cdef^	2.89 ± 0.3^bc^	NA	14.02 ± 0.64^e^	32.17 ± 0.46^b^	36.89 ± 0.34^b^	NA
	T_4_	35.86 ± 0.66^e^	35.93 ± 0.55^cd^	55.68 ± 0.94^b^	NA	1.98 ± 0.29^cdef^	3.61 ± 0.28^abc^	3.77 ± 0.51^ab^	NA	18.26 ± 0.4^d^	35.54 ± 0.12^a^	39.46 ± 0.47^a^	NA
Ambient storage (29±3 °C and 65 ± 2 % RH)	T_0_	52.67 ± 0.52^c^	NA	NA	NA	4.03 ± 0.75^ab^	NA	NA	NA	32.51 ± 0.31^c^	NA	NA	NA
	T_1_	59.19 ± 0.45^b^	NA	NA	NA	4.50 ± 0.54^a^	NA	NA	NA	13.5 ± 0.4^e^	NA	NA	NA
	T_2_	54.04 ± 0.66^c^	NA	NA	NA	2.88 ± 0.49^abcde^	NA	NA	NA	33.51 ± 0.32^bc^	NA	NA	NA
	T_3_	61.21 ± 0.29^a^	NA	NA	NA	3.24 ± 0.47^abcd^	NA	NA	NA	34.63 ± 0.37^b^	NA	NA	NA
	T_4_	44.14 ± 0.7^d^	NA	NA	NA	3.14 ± 0.43^abcd^	NA	NA	NA	33.85 ± 0.43^bc^	NA	NA	NA

Each value is expressed as mean ± SD and different small letters within the same column indicate significant (p ≤ 0.05) differences among various samples. Note: NA = Not applicable; T_0_ = control, T_1_ = 0.5 μL/L 1-MCP, T_2_ = 1.0 μL/L 1-MCP, T_3_ = 1.5 μL/L 1-MCP, and T_4_ = 2.0 μL/L 1-MCP treatment.

The a* value index was increased during storage indicates that the fruits tends to became less green and turning into red. This can be happened due to the degradation of chlorophyll pigment and carotenoids formation [32]. The lowest a* value was recorded during 10 days of storage in fruits treated with 1.0 μL/L 1-MCP (T_2_) at developed storage structure and the value was −1.63 ± 0.50. The negative a* value specify that the fruits preserve its natural green color during 10 days of storage. This negative lowest value revealed that 1-MCP (1.0 μL/L) showed that the best results in delaying the change in fruits color during storage. This might be due the decrease in oxygen and increase in carbon dioxide levels. However, fluctuation of a* was observed during storage, these could be due to the changes in pigmentation when fruits starts to ripen or decay.

### 3.8 Total phenolic content (TPC)

The changes in the TPC in 1-MCP treated and control samples during stored in different conditions were presented in Table 5. During storage, a difference in TPC were detected in the fruits treated with different concentrations of 1-MCP compared to control sample. This difference might be due to the different storage conditions and the application of 1-MCP, which delayed ripening process of fruits. However, the effect of 1-MCP treatment, storage condition, and storage duration on TPC in mango fruits was significant (*p ≤ 0.05*). The TPC was high in control (T_0_) sample and the value was 23.47 ± 4.42 mg GAE/kg FW during 10 days of storage at ambient condition. The samples stored in ambient condition showed higher TPC compared to cold storage and developed storage structure. The trends of TPC in the aspect of storage conditions were as follows: ambient storage > cold storage > developed storage structure during 10 days of storage. However, during 40 days of storage at developed storage structure, the highest TPC was perceived in T_0_ and lowest was found in T_2_; both values were displayed higher compared to previous days of storage. These findings were in agreed with Yuan et al. [8], who found similar behavior in mango fruits treated with 1-MCP. It was observed that during storage the TPC from different concentrations of 1-MCP treated mangoes were significantly lower than that of control samples (*p < 0.05*). The increase of TPC in control sample may be due to the higher moisture loss, oxidative stress and senescence [32]. The loss of moisture during storage leading to dehydration, which can result in concentration effect causing an increase in TPC per unit weight of fruits. Moreover, mango fruits may experience oxidative stress that triggers the production of TPC as a defense mechanism against oxidative stress [39].

**Table 5:** Interaction effects of 1-MCP treatments on changes in the total phenolic content and total flavonoid content in developed storage, cold storage, and ambient storage conditions.

Storage condition	Treatment	Total phenolic content (mg GAE/kg of extract)				Total flavonoid content (mg QE/kg of extract)			
		Storage period (days)				Storage period (days)			
		10	20	30	40	10	20	30	40
Developed storage structure (10±1 °C and 90 % RH)	T_0_	9.32 ± 0.48^fgh^	10.29 ± 2.55^d^	13.36 ± 0.15^de^	17.02 ± 0.27^a^	2.86 ± 0.66^ab^	3.22 ± 0.65^a^	3.23 ± 0.19^a^	3.29 ± 0.45^a^
	T_1_	8.97 ± 9.41^fgh^	10.50 ± 0.55^d^	14.34 ± 0.49^d^	15.93 ± 0.73 ^ab^	2.18 ± 0.80^abc^	2.87 ± 0.63^a^	3.16 ± 0.05^a^	3.41 ± 0.43^a^
	T_2_	8.80 ± 8.76^gh^	10.70 ± 0.94^d^	11.73 ± 0.79^f^	13.16 ± 0.50^d^	2.19 ± 0.58^abc^	2.63 ± 0.79^a^	2.80 ± 0.67^a^	2.80 ± 0.67^a^
	T_3_	10.33 ± 3.37^efg^	14.72 ± 0.54^b^	12.75 ± 0.26^ef^	14.30 ± 0.54 ^cd^	2.31 ± 0.52^abc^	2.57 ± 0.69^a^	2.79 ± 0.85^a^	3.10 ± 0.52^a^
	T_4_	9.38 ± 3.73^fgh^	12.65 ± 0.17^c^	17.58 ± 0.73^c^	15.16 ± 0.61^bc^	2.68 ± 0.29^abc^	2.92 ± 0.71^a^	3.00 ± 0.44^a^	3.29 ± 0.42^a^
Cold storage (3±1 °C and 80 ± 2 % RH)	T_0_	10.48 ± 4.58^ef^	15.30 ± 0.25^ab^	23.71 ± 0.59^a^	NA	2.70 ± 0.39^abc^	2.83 ± 0.49^a^	3.06 ± 0.24^a^	NA
	T_1_	8.26 ± 2.37^h^	16.44 ± 0.70^a^	21.35 ± 0.16^b^	NA	2.12 ± 0.37^abc^	2.14 ± 0.15^a^	2.35 ± 0.64^a^	NA
	T_2_	8.316 ± 3.57^h^	13.73 ± 0.78^bc^	18.57 ± 0.28^c^	NA	1.81 ± 0.16^bc^	2.29 ± 0.79^a^	2.52 ± 0.43^a^	NA
	T_3_	11.69 ± 6.55^e^	12.44 ± 0.09^c^	17.33 ± 0.62^c^	NA	1.74 ± 0.41^c^	2.80 ± 0.62^a^	3.42 ± 0.83^a^	NA
	T_4_	14.00 ± 0.63^d^	16.65 ± 0.48^a^	18.31 ± 0.52^c^	NA	1.77 ± 0.25^bc^	1.87 ± 0.60^a^	2.30 ± 0.68^a^	NA
Ambient storage (29±3 °C and 65 ± 2 % RH)	T_0_	23.47 ± 4.42^a^	NA	NA	NA	3.00 ± 0.67^abc^	NA	NA	NA
	T_1_	18.79 ± 7.69^c^	NA	NA	NA	1.80 ± 0.54^bc^	NA	NA	NA
	T_2_	17.43 ± 4.38^c^	NA	NA	NA	1.63 ± 0.34^c^	NA	NA	NA
	T_3_	18.33 ± 3.28^c^	NA	NA	NA	1.91 ± 0.58^abc^	NA	NA	NA
	T_4_	21.01 ± 0.45^b^	NA	NA	NA	2.13 ± 0.53^abc^	NA	NA	NA

Each value is expressed as mean ± SD and different small letters within the same column indicate significant (p ≤ 0.05) differences among various samples. Note: NA = Not applicable; T_0_ = control, T_1_ = 0.5 μL/L 1-MCP, T_2_ = 1.0 μL/L 1-MCP, T_3_ = 1.5 μL/L 1-MCP, and T_4_ = 2.0 μL/L 1-MCP treatment.

### 3.9 Total flavonoid content (TFC)

Flavonoids are a type of phytochemical that has the ability to combat a variety of degenerative diseases such as cancer, diabetes, hypersensitivity, cardiovascular disease, inflammation etc. [40]. Apart from diseases fighting and health-promoting roles, phytochemicals of fruits can play antioxidant and antimicrobial potential to extend the shelf life of fruits by delaying or inhibiting the growth of microorganisms and lipid oxidation [41]. The TFC are the major compounds presenting antioxidant action, and the value of TFC was seen increasing as the storage period progresses as shown in Table 5. At initial stage of storage, the influence of different storage conditions on TFC in mango fruits was significant (*p ≤ 0.05*). However, a difference observed in TPC and TFC at initial storage, the TPC was higher at ambient condition whereas TFC was found lower irrespective of control treatment. During the entire storage period at developed storage structure, the maximum TFC value 3.29 ± 0.45 mg QE/kg FW was found in control (T_0_) sample at 40 days of storage, which was 2.86 ± 0.66 mg QE/kg FW during 10 days of storage. The control sample exhibited higher TFC compared to treated sample and showed increasing trends for all samples with the increase of storage period. The increase in TFC in control sample might be due to the increased respiration rates related to ripening of fruits [35]. During storage, fruits may experience oxidative reactions which can lead to the polymerization of flavonoids [42], involves the linking of multiple flavonoid molecules resulting in larger and more complex structures. These polymeric forms contribute to the TFC and may increase during storage.

### 3.10 Analysis of regression

**Fig. 3:**
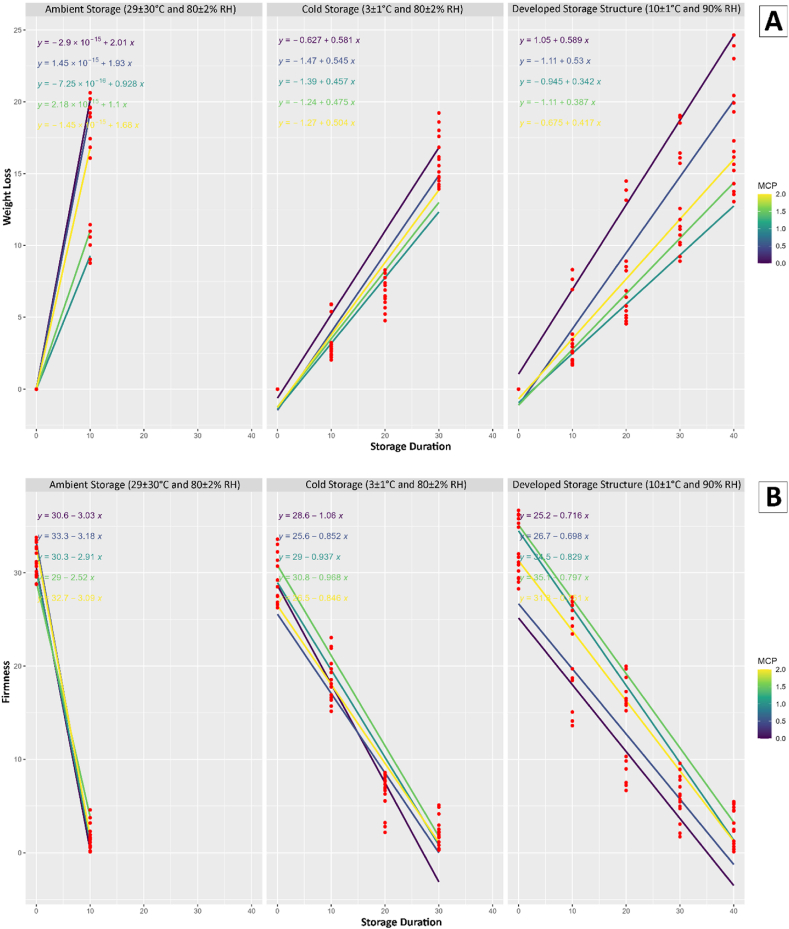

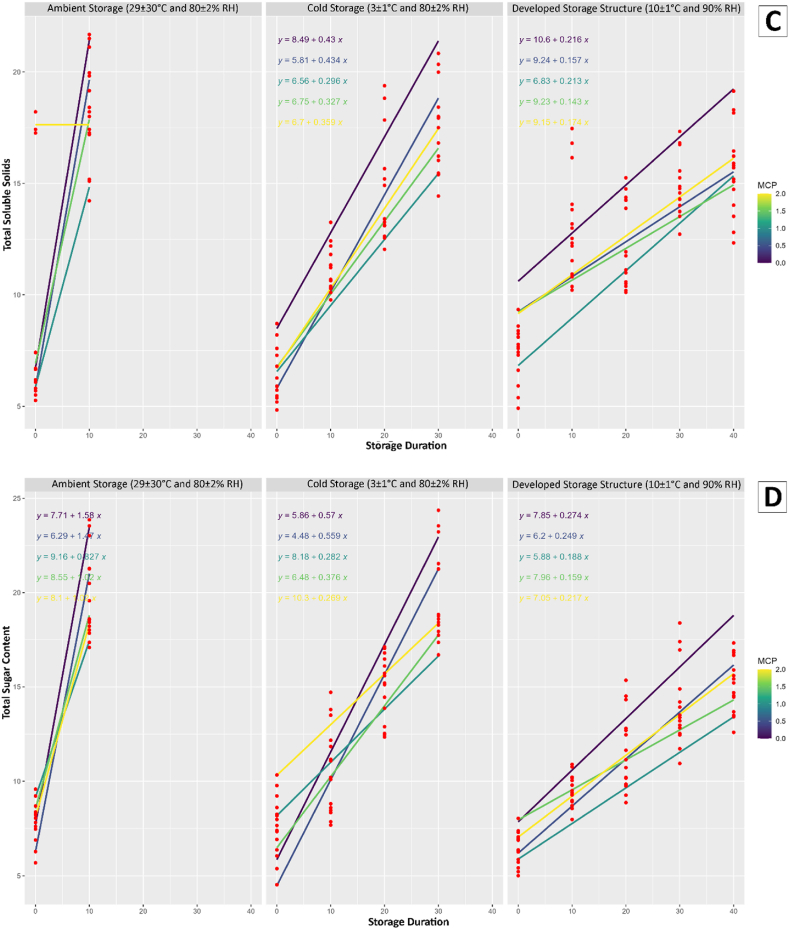

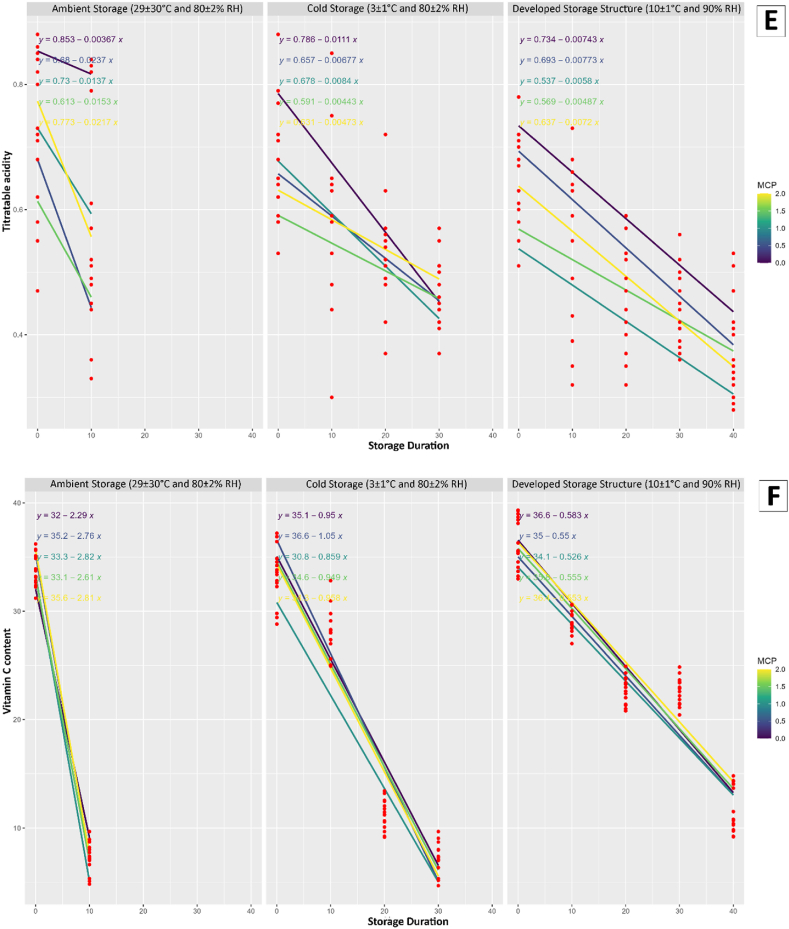

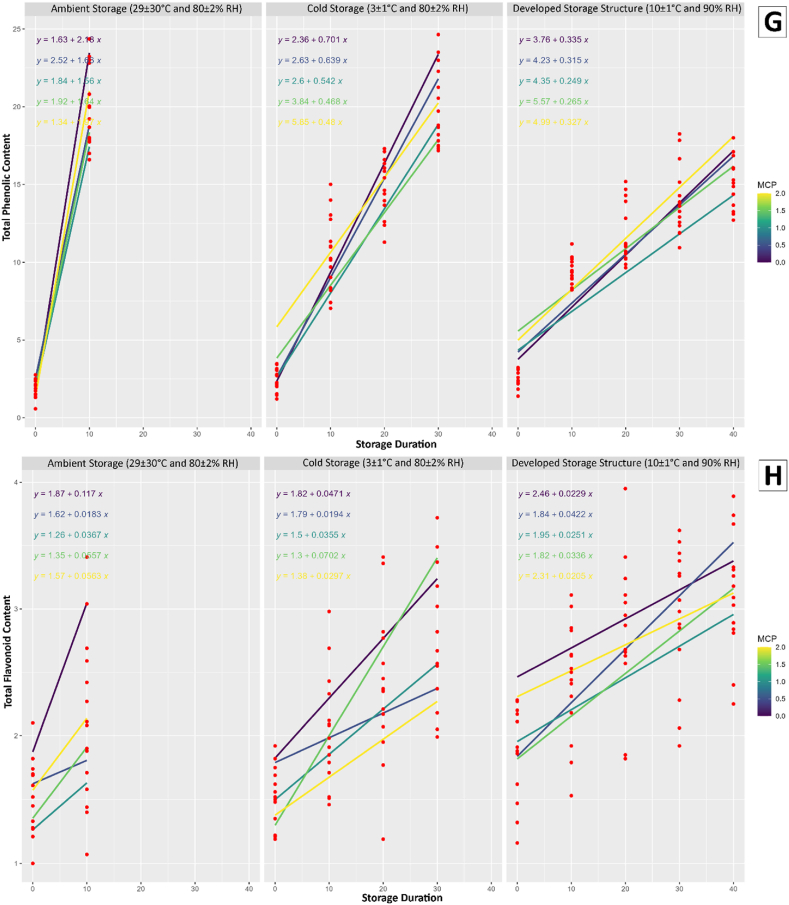
Results of regression analysis; A) Weight loss, B) Firmness, C) Total soluble solids, D) Total sugar content, E) Titratable acidity, F) Vitamin C, G) Total phenolic content, and H) Total flavonoids content for different storage conditions and 1-MCP treatments.

The study conducted a comprehensive regression analysis to investigate the impact of storage duration, storage conditions, and the application of 1-MCP at varying concentrations on multiple quality attributes of mango fruits and the results were presented in Fig. 3 (A-H).

The observed variations in weight loss indicated the effectiveness of the storage conditions and 1-MCP treatment in mitigating the loss of moisture in mango fruits. The present study revealed that the conditions used for developed storage structure and 1.0 μL/L 1-MCP treatment was effectively reduced the loss of fruit weight. The mango fruit treated with 1 % chitosan reduced the loss of weight was mentioned in another study [43]. The firmness of the fruits, a crucial indicator of their textural quality, exhibited fluctuations influenced by the storage conditions and the concentration of 1-MCP. These findings suggest that the choice of storage conditions and treatment application plays a crucial role in maintaining the physical integrity of mango fruits. The study also uncovered insights into the changes in chemical composition during storage. The TSS, TSC, TA, AAC, TPC, and TFC were all found to be influenced by storage conditions and the application of 1-MCP. The analysis of regression highlights the intricate relationship between storage parameters and the biochemical composition of mango fruits. Furthermore, the conditions for developed storage structure showed promising results in line with 1.0 μL/L 1-MCP treatment in preserving the quality attributes of mango fruits compared to ambient and cold storage.

### 3.11 Scanning electron microscopy (SEM)

**Fig. 4:**
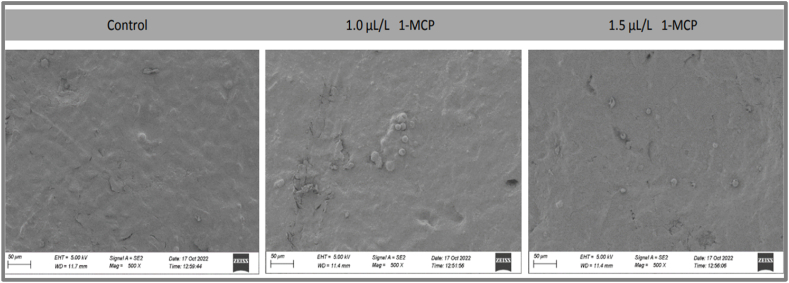
Scanning electron microscopy (SEM) images (50 μM) of the BARI-4 mango fruits' surface treated with 1-MCP at 40 days of storage in developed storage structure (10±1 °C and 90 % RH).

The micromorphology structure of control and 1-MCP treated fruits outer surface were observed by scanning electron microscopy as shown in Fig. 4. SEM images of fruit outer surface showed that 1-MCP treatment had different effect on surface structure. The figure showed that control fruit had roughed surface as compared to treated sample and no granular elements exists. Whereas, images from treated mangoes displayed smooth surface at SEM scanning revealed that 1-MCP treatment act as a barrier around the fruit surface. However, a tiny crack was observed in treated sample at a concentration of 1.0 μL/L that may be due to the natural surface structure related to the maturity degree of fruits.

The highest 1-MCP concentration 1.5 μL/L in this figure represent smoother and less cracking fruit surface. It was evident that 1-MCP treatment delayed ripening of fruits thereby delaying or increasing the formation of cuticular cracking [44]. The postharvest treatment on fruit surface covers the cuticle and blocks the pores creating resistance between internal and external atmosphere of fruits surface [27]. The result suggested that the application of 1-MCP treatment on fruit surface has positive effect on modifying the internal atmosphere by reducing transpiration losses and suppressing the respiration rate.

### 3.12 Storage life of 1-MCP treated and control mango samples

**Fig. 5:**
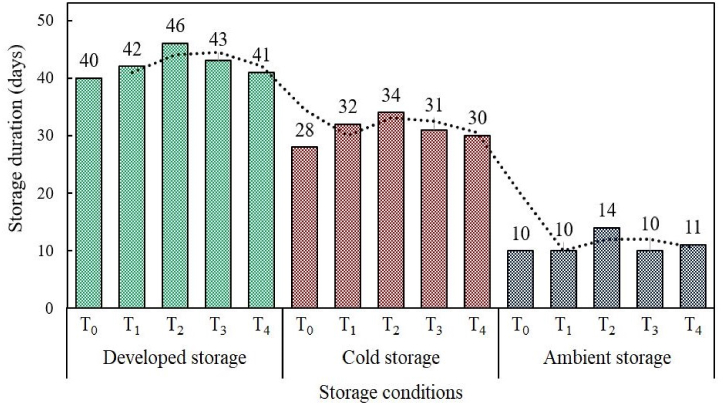
Storage life of mango fruits treated with different concentrations of 1-MCP. Note: T_0_ = control T_1_ = 0.5 μL/L 1-MCP, T_2_ = 1.0 μL/L 1-MCP, T_3_ = 1.5 μL/L 1-MCP, T_4_ = 2.0 μL/L 1-MCP treatment.

Storage life plays an important role in the marketplace as a commercial quality indicator of fruits [45]. Storage life (days) of treated and control fruit samples was measured in this study at the limit of acceptability and presented in Fig. 5. The storage stability was assessed based on the visual appearance and texture of fruits, with the presence of any visible signs of spoilage serving as crucial indicators for acceptability. A limit was fixed on these indicators to determine the acceptability of the fruits. From the figure, it can be summarized that storage conditions and different concentrations of 1-MCP treatment influenced on the storage life of mango samples. The lower concentrations of 1-MCP treatment exhibited higher storage life compared to control and higher concentrations of 1-MCP treatment among all the three storage conditions. Moreover, the developed storage structure presented higher storage life compared to other storage conditions and the trends were in order of developed storage structure > cold storage > ambient storage.

The 1-MCP treated samples especially T_2_ (1.0 μL/L) exhibited highest storage life and the maximum values were 46 days, 35 days, and 14 days of storage in developed storage structure, cold storage and ambient storage respectively. Whereas, the control sample showed maximum storage life in developed storage structure, cold storage and ambient storage were 40 days, 28 days, and 10 days respectively. The storage life of 1-MCP (1.0 μL/L) treated samples stored in developed storage structure displayed desirable performance compared to other treatments and storage conditions. These results were in consisted with the findings of Wongmetha et al. [46], who found the storage life of mango fruit treated with 1-MCP was 28 days at cold storage (10 °C). Earlier studies also revealed that the treatment with 1-MCP was potentially prolonged the storage life of ready-to-eat kiwifruit at ambient temperature 23 ± 1 °C [47]. Postharvest 1-MCP treatments have barrier properties that inhibit ethylene biosynthesis, which retards the respiration rate, retain firmness and delay fruit ripening which in turn increases the storage life of fruits [48]. Advanced microbiological study suggested that 1-MCP suppressed anthracnose, which is one of the most postharvest diseases in mangoes; by directly inhibiting spore germination and mycelial growth of *Colletotrichum gloeosporiodies* [49]. Thus, provided a promising strategy for postharvest disease control and enhancing storage life of mango fruits.

## 4 Conclusion

The present study revealed that, different concentrations of 1-MCP treatment and storage conditions aided in extending the storage life of mango (var. BARI-4). The quality of fruits was assessed at different intervals exposed that, all the physicochemical and bioactive properties were significantly influenced by 1-MCP treatments and storage conditions. The developed storage structure (10±1 °C and 90 ± 1 % RH) and 1-MCP (T_2_ = 1.0 μL/L) retained physiological weight loss, textural firmness, and delayed the climacteric ripening to a greater extent compared to other storage conditions and 1-MCP concentrations. The storage conditions employed in developed storage structure with 1-MCP treatment extended the storage life of mango fruits about 40 days, whereas cold storage (3±1 °C and 80 ± 2 % RH) and ambient storage (29 ± 3^0^C and 85 ± 2 % RH) were able to preserve about 30 days and 10 days respectively. These results suggested that storage conditions used in developed storage structure and 1-MCP treatments inhibited the softening and delayed ripening of BARI-4 mango. Therefore, 1-MCP (1.0 μL/L) and conditions for developed storage structure could be used as a novel postharvest treatment and storage condition respectively in commercial application for prolonging the storage life of mango fruits.

## Data availability statement

The data that support the findings of this study are available from the corresponding author upon reasonable request.

## CRediT authorship contribution statement

**Mustafa Kamrul Hasan:** Writing – original draft, Methodology, Formal analysis, Data curation, Conceptualization. **Asraful Alam:** Writing – original draft, Methodology, Formal analysis, Data curation, Conceptualization. **Md. Rakibul Islam:** Writing – review & editing, Writing – original draft, Visualization, Validation, Methodology, Conceptualization. **Md. Akhtaruzzaman:** Writing – review & editing, Supervision, Project administration, Funding acquisition, Conceptualization. **Mrityunjoy Biswas:** Writing – review & editing, Supervision, Project administration, Funding acquisition, Conceptualization.

## Declaration of competing interest

The authors declare no potential conflict of interest.
